# Steering of carbon fiber/PEEK tapes using Hot Gas Torch-assisted automated fiber placement

**DOI:** 10.1177/08927057211067962

**Published:** 2022-02-14

**Authors:** Aadhithya Rajasekaran, Farjad Shadmehri

**Affiliations:** 1 Department of Mechanical, Industrial and Aerospace Engineering, 5618 Concordia University, Montreal, Quebec, Canada; 2 Research Center for High Performance Polymer and Composite Systems (CREPREC), Montreal, Quebec, Canada

**Keywords:** Automated fiber placement, fiber steering, variable angle tow, thermoplastic composite, carbon fiber/polyether ether ketone, lap shear test

## Abstract

In-situ manufacturing of thermoplastic composites using Hot Gas Torch (HGT)-assisted Automated Fiber Placement (AFP) has the potential to produce laminates in an efficient manner by avoiding a secondary process, like autoclave consolidation. One of the advantages of AFP technique is its capability to steer fiber path and to manufacture Variable Angle Tow (VAT) laminates which have shown to have improved mechanical performance. This study investigates the process parameters that affect steering of carbon fiber reinforced thermoplastic tapes (AS4/polyether ether ketone) using an HGT-assisted AFP machine. The effect of the steering radius, laydown speed, number of repasses, and substrate angle on the geometry and bond strength of steered tape was investigated through observation and testing. A modified lap shear test was devised and used to study the bond strength between the steered tape and the substrate and the results were compared with autoclave treated samples which served as a reference. It was found that with a decrease in the steering radius of the tape, there was a decrease in the tape width and an increase in the tape thickness. A significant reduction in the steering-induced defects was observed at higher laydown speeds where the defects were intermittent unlike in the case of lower laydown speeds. Performing a repass over the steered tape smoothed some of the tape defects caused by steering. Furthermore, the lap shear strengths of the steered tapes were found to be functions of laydown speed and substrate angle.

## Introduction

**Figure 1.**
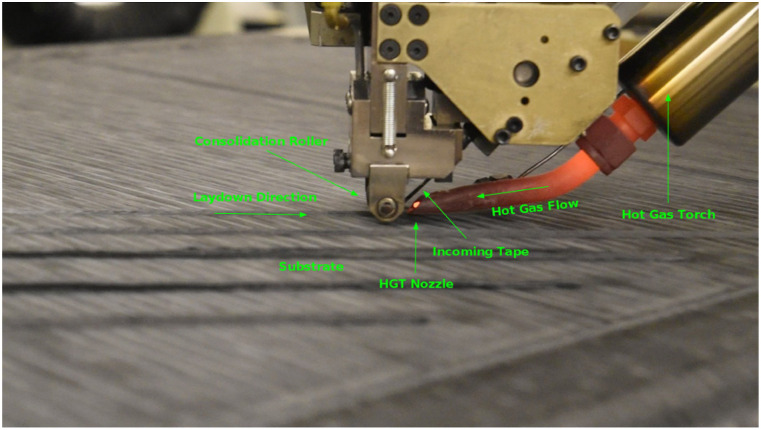
Hot Gas Torch-assisted Automated Fiber Placement.

There is a large demand for automated manufacturing processes in the production of fiber-reinforced composites; automation makes it possible to have a reliable and repeatable manufacturing process which is not dependent on operators’ skills. Among automated manufacturing techniques, Automated Fiber Placement (AFP) technique attracts interest from aerospace and automotive industries and provides a new approach to the manufacturing of large-scale complex composite parts. Thermoplastic composites, in comparison with thermoset composites, have a unique advantage with the possibility of in-situ consolidation; thus, avoiding secondary processes such as autoclave treatment which leads to significant cost/energy savings. They are also reworkable, have high temperature performance, recyclable, show high impact resistance, and do not require freezing temperatures for storage.^1–6^ The HGT-assisted AFP is one such in-situ manufacturing process of thermoplastic composites.^7^ In the HGT-assisted AFP a prepreg thermoplastic tape is fed to the roller and is heated above its melting temperature by a hot gas (e.g., nitrogen). The tape is then laid down on the substrate and bonded to it by a compaction force applied by a compaction roller. The schematic of the principle used in HGT-assisted AFP is shown in Figure 1. Laser-Assisted Tape Placement (LATP) is very similar technique where a Laser is used as the heat source instead of a heated gas like nitrogen.^8–10^

An important aspect of this technique is its ability to manufacture Variable Angle Tow laminates (VAT). VAT laminates are used to streamline the stiffness of a component as by placing fibers along the direction of maximum stress.^11–14^ A simple example of this is the use of VAT laminates to remove the stress concentration around the hole in a panel, which was explored in a study by Setoodeh et al.^11^ A study performed by Gürdal et al. studied the effect of VAT laminates on the in-plane and the buckling response of the laminate.^13^ They demonstrated the flexibility available to a designer when using VAT laminates to tailor the laminate for high plane stiffness or buckling load as per their requirement. The use of VAT laminates also improves the first-ply failure, compression, and buckling properties.^12^ Another study performed by Rouhi et al. showed that a cylinder produced by steering the fibers can improve the bending-induced buckling performance by up to 18*%*.^14^ Nevertheless, there are common defects associated with manufacturing of VAT laminates including tow buckling, tow folding, gaps, and overlaps^13,15^ which mainly affect adversely the mechanical performance of these laminates. For example, tow buckling and tow folding defects reduce the bonding strength between the steered tow and the substrate.^15^ Thus, understanding the effect of AFP process parameters and steering radii on creation of these defects and their subsequent effects on geometrical and mechanical properties of the VAT laminates is essential.

Apart from in-situ consolidation of thermoplastic composites using AFP, there are other automated methods of manufacturing VAT laminates, namely, Prepreg Thermoset Tape Placement and Dry Fiber Placement. Filament winding, a type of dry fiber placement, can be used to produce variable angle laminates, but it is usually restricted to closed shapes such as pipes and pressure vessels.^16^ Thermoset prepreg tape placement is most widely used to produce VAT laminates. The steering radii of these tapes are restricted due to the resin/matrix of the prepreg tape confining the fibers and preventing it from following the guide curve without any buckling or stretching. Defects such as tow kinking were observed by Wu et al. during the steering process of cylindrical shells.^17^ Studies have shown that a minimum steering radius of 635 *mm* is achievable by using thermoset prepreg tapes.^18^

However, the thermoset tape placement process is not in-situ, *that is,* the curing of the resin does not take place simultaneously as the tape is laid down but requires an energy and time-consuming secondary process of curing in an autoclave. Dry Fiber Placement (DFP) avoids the defects observed in the thermoset prepreg tape placement such as tow kinking and tape folding as the fibers are not confined by the resin allowing them to bend and shear to conform more accurately to the guiding curve.^19^ Due to this reason, radius of 400 *mm* is achievable using this process.^20^ To secure the fibers in position, binder and/or veil (thermoplastic-based or epoxy-based) may be added to the dry fiber tape which will be melted during deposition and will fix the fibers in place. However, DFP requires additional steps of resin infusion and autoclave curing; thus, increasing the time required for the process.

Continuous Tow Shearing (CTS) is a type of dry fiber placement process where it uses the advantage of dry fibers being able to shear to conform to a curve without buckling.^21^ However, it also attempts to stick the fibers and infuse resin in them by using a separate resin film that is fed to the roller along with the dry fibers. While the use of the resin film does improve the process efficiency, it still results in a poor infusion leaving a lot of regions with dry fibers. This technique also induces thickness variations as the dry fibers tend to slide over one another while they shear and in extreme cases results in fiber bulges.^21^

The ability of thermoset tapes to produce VAT laminates and the influence of defects on part performances have been explored extensively in several studies.^22–25^ In comparison, the number of studies on the production of VAT laminates using in-situ consolidation of thermoplastic prepreg tapes are very limited.^7,15,20^ In a study by Lamontia et al.,^7^ they manufactured several components like pipes, panels, rings etc., by using in-situ consolidation of AS4-polyether ether ketone (PEEK) tapes with a hot gas torch (HGT). They also briefly explored the ability of the HGT-assisted AFP to steer AS4-PEEK tapes and succeeded in placing tows with a minimum radius of 1270 *mm* without any noticeable defects due to steering. Another recent study by Clancy et al.^15^ investigated the ability of a Laser-Assisted Tape Placement (LATP) process to steer CF-PEEK tapes and the influence of the process parameters on the bond strength. The process parameters considered by them were the steering radius of the tape and laydown speed. They succeeded in producing steered tapes up to a minimum radius of 400 *mm* with minimal or equal number of defects in comparison with the thermoset counterparts. The bond consolidation was studied with the help of a microscope and a novel peel test was devised to test the bond strength of the steered tape with the substrate. The microscopy showed good bond consolidation overall except at the edges which suffered due to the buckling and tape folding defects. The quantitative results of the mechanical testing were not conclusive as the failure modes of the peel test were not as expected. While the quantitative values were unreliable from the mechanical testing, they qualitatively concluded from the trends observed that peel strength decreases with increasing laydown speed while it increases with increasing steering radius.^15^

This paper studies steering of CF/PEEK tapes using HGT-assisted AFP. HGT has some advantages over laser heat source including more distributed and effective heating in joining areas, it can be used in processing glass-fiber based composites, and there are no safety hazards related to use of laser. The effects of the steering radii, laydown speed, and re-consolidation pass (referred to as “repass” in the paper) on the dimensional variation of the steered tape on a flat surface are studied and the consolidation of the steered tape was observed through optical microscopy. Repass is an in-situ treatment which refers to the application of heat and pressure using the AFP head to an already placed tape, without the addition of new material.^26^ In addition, the effects of steering radii, laydown speed, and substrate fiber angle on the bond strength of the steered tape are studied**.** In order to overcome the issue related to the failure modes of peel test mentioned in Ref. 15, a specially devised lap shear test is proposed to test the bond strength of a steered and in-situ consolidated tape. Straight samples made by AFP and then reconsolidated inside the autoclave are used as references and their bond strengths are compared with those of AFP in-situ consolidated samples.

## Materials and manufacturing setup

This study uses the HGT-assisted AFP to perform the in-situ consolidation of carbon fiber/PEEK composite tape. The thermoplastic AFP head system supplied by Trelleborg is mounted on to a Kawasaki articulated arm robot which has 6 axis and 125 *kg* payload. A flat aluminum mandrel was used to perform all the trials. The carbon fiber (AS4)/PEEK (APC-2) tape provided by Solvay is used in this study. It has a width of 6.35 *mm* (0.25*in*), a thickness of 0.163 ± 0.017* mm*, a fiber volume fraction of 60, and a glass transition temperature of 143°*C* (289°*F*). Roughness (arithmetic mean deviation from the mean (Ra)) of the tape perpendicular to fiber direction after processing with no repass and one repass was measured to be 40.7 ± 3.57 *μm* and 6.2 ± 0.41 *μm*, respectively.^26^ Nitrogen was used as the hot gas in the HGT-assisted AFP process at a temperature of 875°*C* and a flow rate of 0.06 *m*^3^/min. A steel compaction roller with a diameter of 12.7* mm*(0.5*in*) and a width of 17.78* mm*(0.7*in*) was used in this study as it can withstand very high temperatures without degrading or experiencing large dimensional changes. The consolidation force used in the manufacturing of substrate and samples was 266.89 *N*. Lower consolidation forces (178*N* − 222*N*) and hot gas temperatures (750°*C* − 850°*C*) were tested during preliminary trials, but there was a consistent fiber roll up issue when these parameters were used. After increasing the consolidation force to 266.89* N* and the temperature to 875°*C*, this issue was resolved.

**Figure 2.**
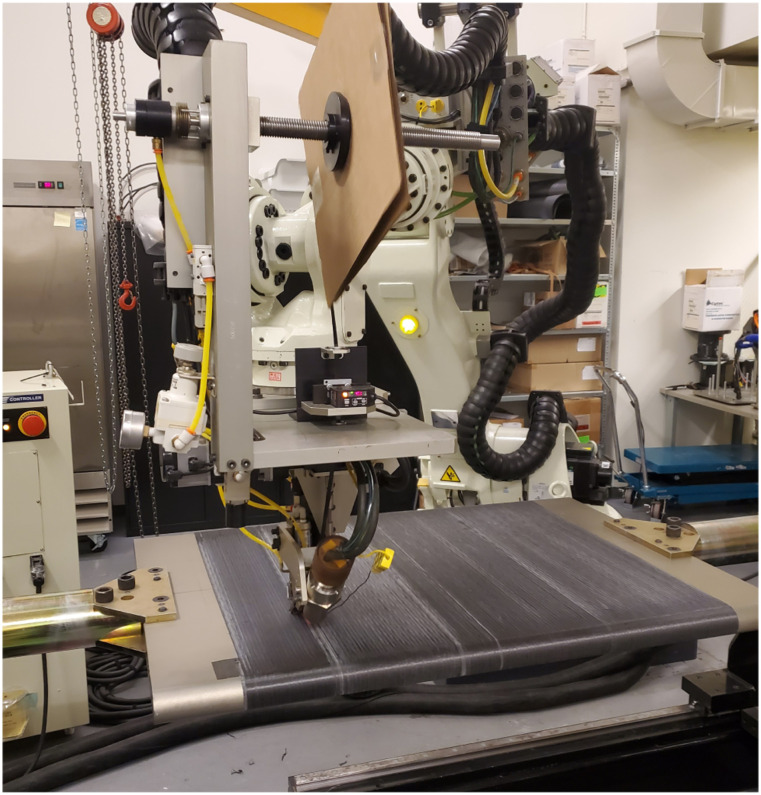
Substrate preparation using Hot Gas Torch Automated Fiber Placement setup.

A flat laminate of configuration [0_4_] was manufactured using the HGT-assisted AFP as a substrate on a flat mandrel. The first two layers were wrapped around the mandrel while the next two layers were laid down in a rectangular area on the top of the previous two layers as shown in Figure 2.

### Reference sample manufacturing using autoclave

**Figure 3.**
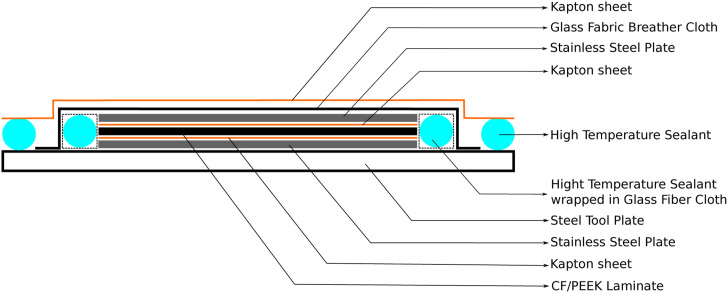
Vacuum bag scheme used for reference samples.

**Figure 4.**
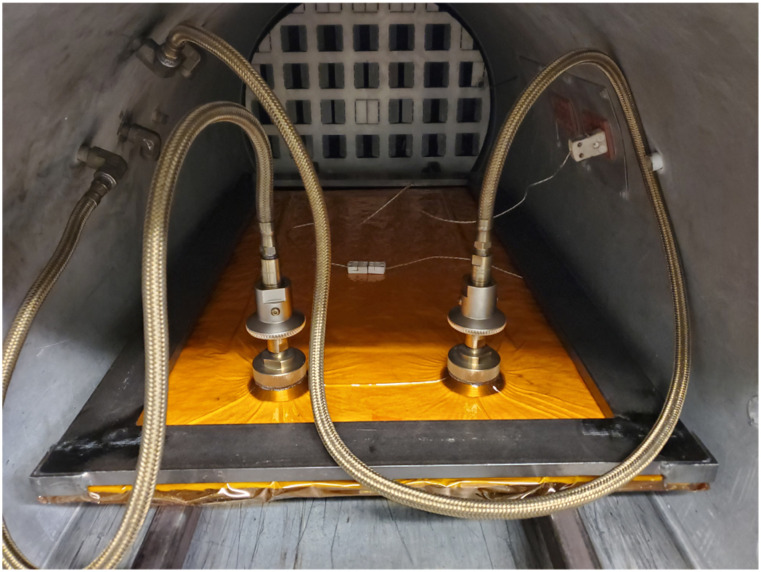
Reference samples prepared in the autoclave.

For comparison purpose, some samples were manufactured first by using the HGT-assisted AFP and then reconsolidated in an autoclave to serve as a reference for bond strength evaluation through mechanical testing (i.e., modified lap shear test). Flat substrate panels were made using the HGT-assisted AFP similar to the abovementioned procedure and cut to size. On this substrate, both straight and steered tapes were laid down and the gauge area was limited to a height of 6.35 *mm* like all other samples. Since these samples will be re-consolidated in an autoclave before testing, laying down only a single tape, straight or steered, will be insufficient and result in an undesired failure mode (fiber breakage). After some trial and error, it was found that a total of six tapes will be safe and reliably result in the desired failure mode (cohesive failure, see the section *Mechanical Testing*). The samples were then prepared to be placed in the autoclave by vacuum bagging them. Ten samples were processed at once by placing them next to each other. Thin strips of Kapton® HN general-purpose polyimide film were used to prevent the two halves of each substrate from fusing together. The samples are sandwiched between two stainless steel caul plates that are covered by Kapton® films. The Kapton® films were double coated with Frekote® 770-NC release agent to facilitate easy removal of the laminate. Since PEEK requires a high processing temperature of 391°*C*(735°*F*), care was taken so that everything used in the vacuum bagging process was able to withstand that temperature. The complete vacuum bag scheme is shown in Figure 3. After sealing the vacuum bag, a steel frame was placed on the top of the sealant, as shown in Figure 4, to prevent any leaks due to possible sealant failure at high temperature.

**Figure 5.**
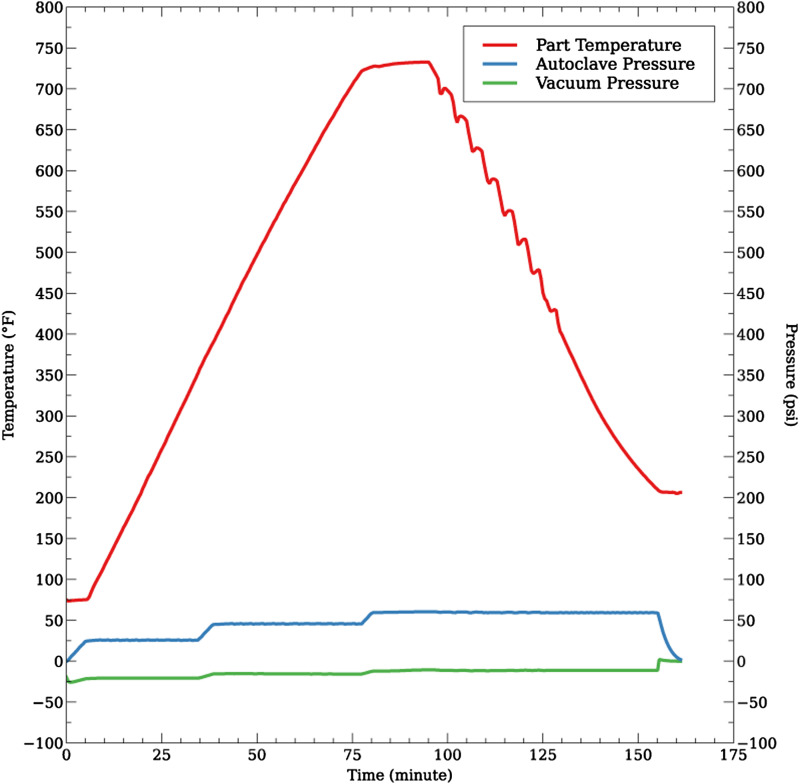
Autoclave thermal cycle.

The sample was then placed in the autoclave and a vacuum of −81.273 *kPa*(−24 *inHg*) was applied to it. The autoclave was then run up to a maximum temperature of 399°*C*(750°*F*) at a pressure of 266.89 *N*(60*lbs*) for a dwell time of 20 min and then cooled down. The thermal cycle used is shown in Figure 5.

## Design of Experiment

The parameters investigated in this study are the steering radii, laydown speed, and number of repass. Steered tapes with different radii from 1000 *mm* to 200 *mm* were manufactured while the laydown speed of 2.54 *cm*/*s* and 0 repass were kept as constants to understand the effect of steering radii on the geometry and consolidation. Similarly, for laydown speeds varying from 2.54 *cm*/*s* to 12.7 *cm*/*s* (1*in*/*s* to 5*in*/*s*), the steering radius of 400 *mm* was chosen to be constant. And for the effect of repass was evaluated on three different steering radii while keeping the speed at 5.08 *cm*/*s* (2*in*/*s*). Table 1 summarizes all samples that were manufactured for testing the effects of different process parameters.

**Table 1.** Design of experiment.

Sample no	Radius(mm)	Laydown speed (cm/s)	No. of repass
1–15	1000, 800, 600, 400, 200	5.08	0
16–30	400	2.54, 5.08, 7.62, 10.16, 12.7	0
31–39	600, 400, 200	5.08	0, 1, 2

HGT temperature: 875°*C*, HGT flow rate: 0.06 *m*^3^/min, and Compaction Force: 266.89 *N*.

**Figure 6.**
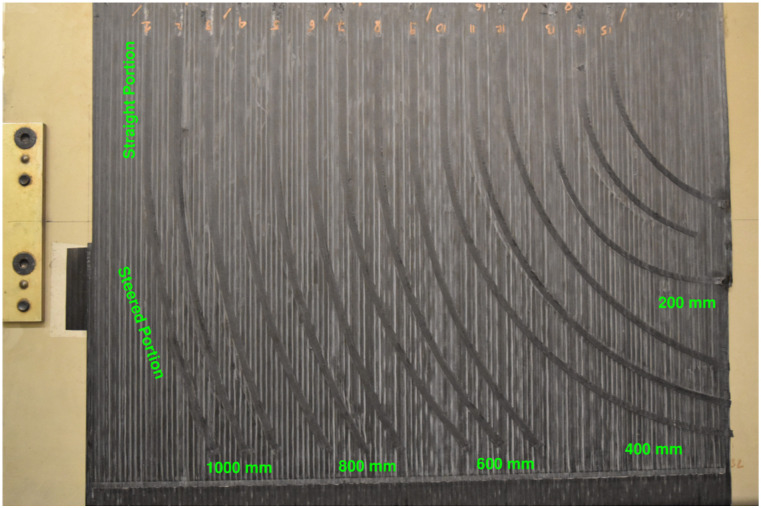
Tapes with different steering radii.

All the samples were designed with an initial straight portion before they were steered at radii as shown in Figure 6. This was to ensure good consolidation with the substrate and to compare the results of analysis performed on the steered tapes with the straight tapes that were laid down with the same process parameters.

## Sample evaluation

### Geometrical analysis

A geometric analysis was carried out on the steered tape to understand how the width and the thickness of the steered tape change in comparison to a straight tape that was laid down with identical processing parameters. Furthermore, measurements were taken to study the percentage of length of the steered tape affected by steering-induced defects such as tape buckling and tape folding.

The average width for the straight portion of the tape was obtained by taking 30 measurements from the three instances in total using a “Mitutoyo” digital Vernier caliper. Similarly, the average thickness was obtained using a “Mitutoyo” digital micrometer by taking 30 measurements from the three instances. This was repeated for every sample of different radii, laydown speed, and number of repass.

Initially, the thickness measurements were taken along with the substrate thickness. Then the average thickness of the substrate alone was measured and found to be 0.639 9 ± 0.0 249* mm***.** This average substrate thickness was obtained by taking 50 measurements at random locations of the substrate. The thickness of the steered layer alone was obtained by subtracting the average thickness of the substrate from it. To calculate the percentage of the tape affected by steering-induced defects, total arc lengths were calculated from the Solidworks design file and the defective arc lengths were measured by small differential lengths of 2 mm using the Vernier Caliper, in cases where the final measurement was less than 2 mm the exact measure was taken.

### Microscopic analysis

Optical microscopy was performed to see the effect of steering, steering-induced defects, and process parameters on the bond quality and fiber distribution. Samples were cut into equal widths using a diamond edge saw. They were placed in a holder, and thin epoxy resin (DER 324) supplied by Anamet was added until the samples were just submerged. The resin was let to cure for 24 h at room temperature and then was post-cured at 100°*C* for 1 h. Samples were then sanded to expose the composite laminate’s cross-section and to obtain a translucent finish. They were then polished using 9 *μ*m and 3 *μ*m fine papers and diamond suspensions to obtain the clear transparent finish required for microscopy.

### Mechanical testing

**Figure 7.**
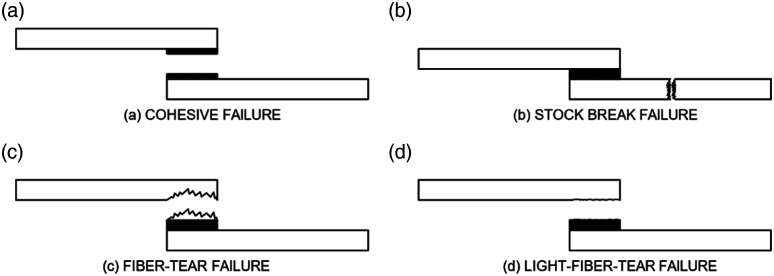
Lap shear test failure modes.^29^

A specially devised lap shear test was used to test the bond strength between the steered tape and a flat substrate prepared using the HGT-assisted AFP. This test is devised based on the Standard lap shear test (ASTM D5868-01) which is primarily for unidirectional Fiber-Reinforced Plastic (FRP) laminates.^27^ The modified test was devised to be used for a single tape that was steered on a flat substrate. The gauge area of the proposed lap shear test is approximately 60 *mm*^2^(0.093*in*^2^) when compared to the recommended gauge area of 645.16 *mm*^2^(1*in*^2^) ASTM D5868-01 standard. The reason for selecting a smaller gauge area is to avoid stock-break (fiber breakage) failure mode which is not a desired failure mode in lap shear test. The gauge area was calculated such that the resulting failure mode would be Light-Fiber-Tear (LFT) or Cohesive failure as shown in Figure 7.^28^

**Figure 8.**
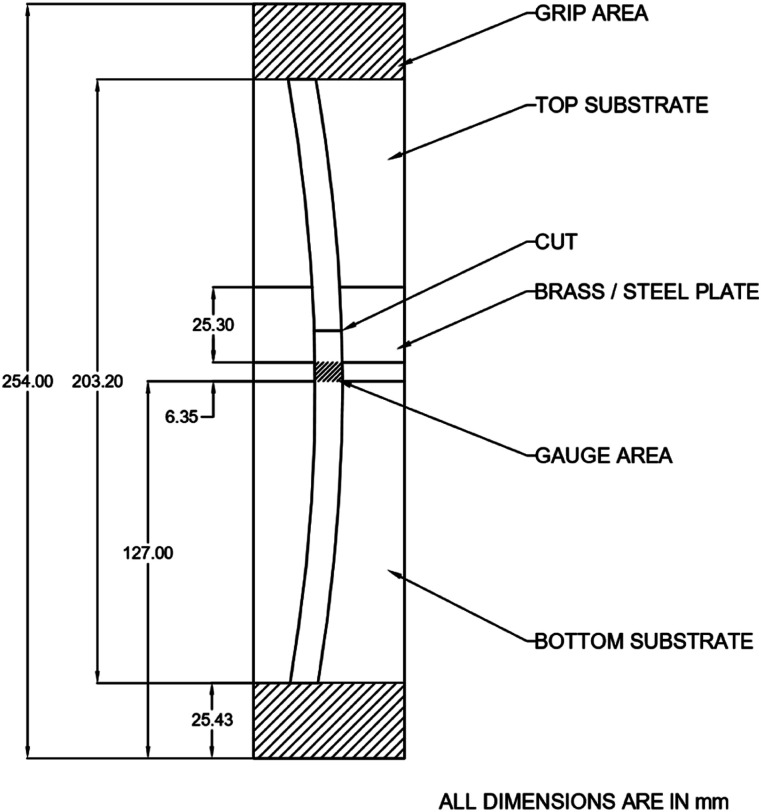
Modified lap shear test schematic.

**Figure 9.**
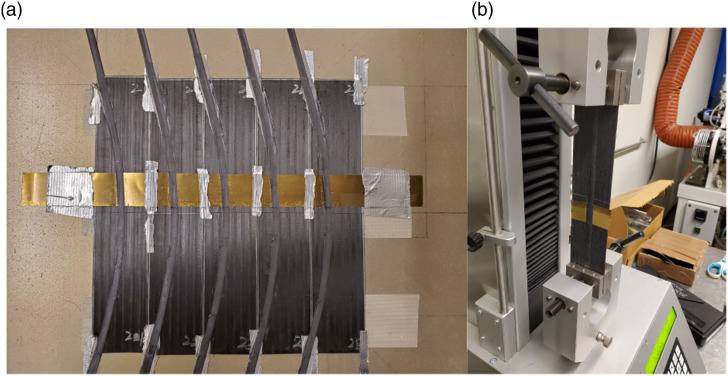
Modified lap shear test.

Flat substrate panels were manufactured using the HGT-assisted AFP with the configuration of [0_4_]. These panels were then cut into 127 *mm* X 50.8 *mm* pieces. Two of these pieces were then affixed on the mandrel using double-sided tape to form a 254 *mm* X 50.8 *mm* substrate for a single sample. To limit the length of the gauge area to 6.35 *mm* (0.25*in*), a 25 *mm* wide and 0.125 *mm* thick brass plate was placed to prevent the steered tape from sticking to the substrate. The brass plate was held in place using duck tape on the sides of the substrate. Bonding the brass plate to substrate with an adhesive is not advised as the adhesive would melt under the high temperature of the HGT and will affect the bond between the tape and substrate. The tape is then steered over the substrate at the desired radius and speed. After steering, the tape over the brass plate is cut so that bond strength at the gauge area can be tested by applying a tensile load. Figures 8 and 9 show the schematic and preparation of the sample for the specially devised lap shear test which is tested under tensile load using a Universal Testing Machine (UTM).

## Results and discussion

### Visual inspection

Steering a flat thermoplastic prepreg tape can result in some inherent defects such as buckling along the inner edge due to compression and tape folding along the outer edge due to excessive tension. A visual inspection was carried out to observe such defects caused by steering and to assess the quality of the samples manufactured using different process parameters.

#### Effect of steering radius

**Figure 10.**
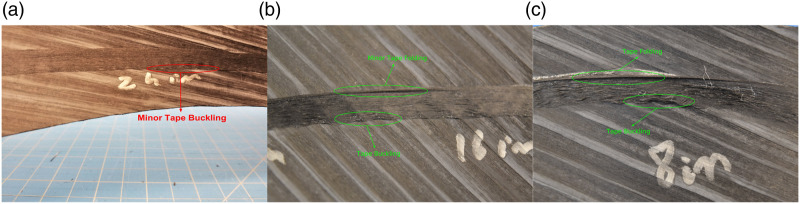
Visual inspection of different steering radii.

From visual observation of sample number 1 to 6 (Table 1), it was clear for radii equal to or larger than 800 *mm* no steering-induced defects like buckling and tape folding were observed. At these radii, the deviation from a straight path is not large enough to produce defects like tape folding due to tension and/or tape buckling due to compression at the edges of the tape. Observation of sample number 7 to 9 (Table 1) with a steering radius of 600 *mm* showed some minor tape buckling on the inner radius of the tape while no tape folding on the outer edges of the tape was observed as shown in Figure 10(a).

From the observation of sample number 10 to 12 (Table 1) with a steering radius of 400 mm, the buckling was prominent and observed all along the inner edge of the samples. Some hints of tape folding were also observed on the outer edge of the tape at this radius, shown in Figure 10(b). Sample number 13 to 15 (Table 1) with the smallest radius of 200 mm suffered from both tape buckling at the inner radius and tape folding of the outer radius as shown in Figure 10(c).

#### Effect of laydown speed

**Figure 11.**
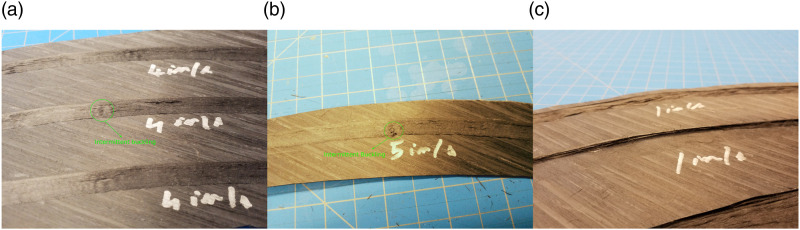
Samples with different laydown speeds steered at 400* mm*. (a) Intermittent defects at 4in/s, (b) intermittent defects at 5in/s, and (c) poor quality at laydown speed of 1in/s.

To understand the effects of laydown speed on the steering of AS4/PEEK tape, different speeds from 2.54 *cm*/*s* (1*in*/*s*) to 12.7 *cm*/*s* (5*in*/*s*) (Table 1) were tested on a steering radius of 400 *mm*. This was because the tape with 400 *mm* steering radius showed both continuous fiber buckling and some hints of tape folding. By varying the laydown speed at this radius, it would be easier to observe how it affects the steered tape. The temperature and compaction force were kept constant at 875°*C* and 266.89 *N* (60*lbs*), respectively. Observation of sample number 19 to 21 and 21 to 24, with laydown speeds of 5.08 *cm*/*s* (2*in*/*s*) and 7.62 *cm*/*s*(3*in*/*s*), respectively, showed continuous tape buckling and some hints of tape folding. This was consistent with the observation made for the samples with 400 *mm* steering radius in the section *Effect of Steering Radius*. However, a significant change to this trend was observed in samples with laydown speeds higher than 7.62 *cm*/*s* (3*in*/*s*). Sample number 24 to 27 and 27 to 30, with laydown speeds of 10.16 *cm*/*s*(4*in*/*s*) and 12.7 *cm*/*s* (5*in*/*s*), respectively, showed only intermittent tape buckling and no tape folding. In case of the laydown speed of 12.7 *cm*/*s* (5*in*/*s*), it was found that 14.26*%* of the tape was affected by buckling defects. While in the case of 10.16 *cm*/*s*(4*in*/*s*), only 6.2*%* of the tape was affected by buckling. This is very different when compared to cases at lower speeds with continuous buckling (e.g., Steering radii from 400 *mm*) where close to 100*%* of the tape is affected by buckling. This effect was probably due to the resin not having enough time to melt completely and to bond with the substrate allowing the fibers to shear and not to buckle. Figure 11(a) and (b) show the intermittent defects observed.

On the other hand, the low speed of 2.54 *cm*/*s*(1*in*/*s*) produced very poor results. This was probably because of the combination of a high temperature of 875°*C* and low speed of 2.54 *cm*/*s*(1*in*/*s*) which allowed a large amount of heat to be transferred to the tape. Due to this, the resin melted completely, and its viscosity was also reduced to the extent that the fibers had less support during the steering process and were allowed to roll up along the tape width as shown in Figure 11(c). Furthermore, some matrix degradation could have happened due to exposure to high temperature in a longer period of time which led to some matrix-poor areas.

#### Effect of repass

**Figure 12.**
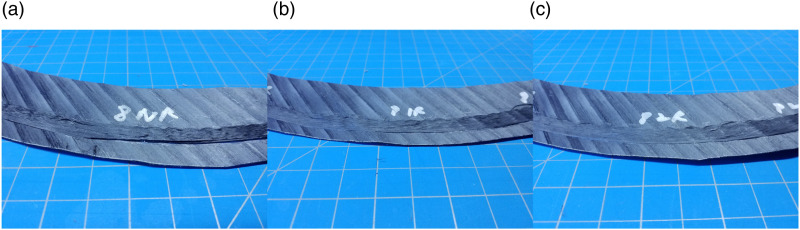
Visual inspection of the effect of repass.

Repass was considered as a separate processing parameter to see if it can ameliorate defects caused by the steering of the tape. Observation showed that a repass is very effective in smoothing over any fiber pull up caused by the buckling of the steered tape. However, it must be noted that repass failed to completely smooth over the tape folding observed in the radii of 400 *mm* and 200 *mm* and may have even caused the folded tape to break at certain locations. Figure 12(a)–(c) shows the effect of repass on the radius of 200 *mm*.

### Geometrical analysis

#### Effect of steering radius

**Figure 13.**
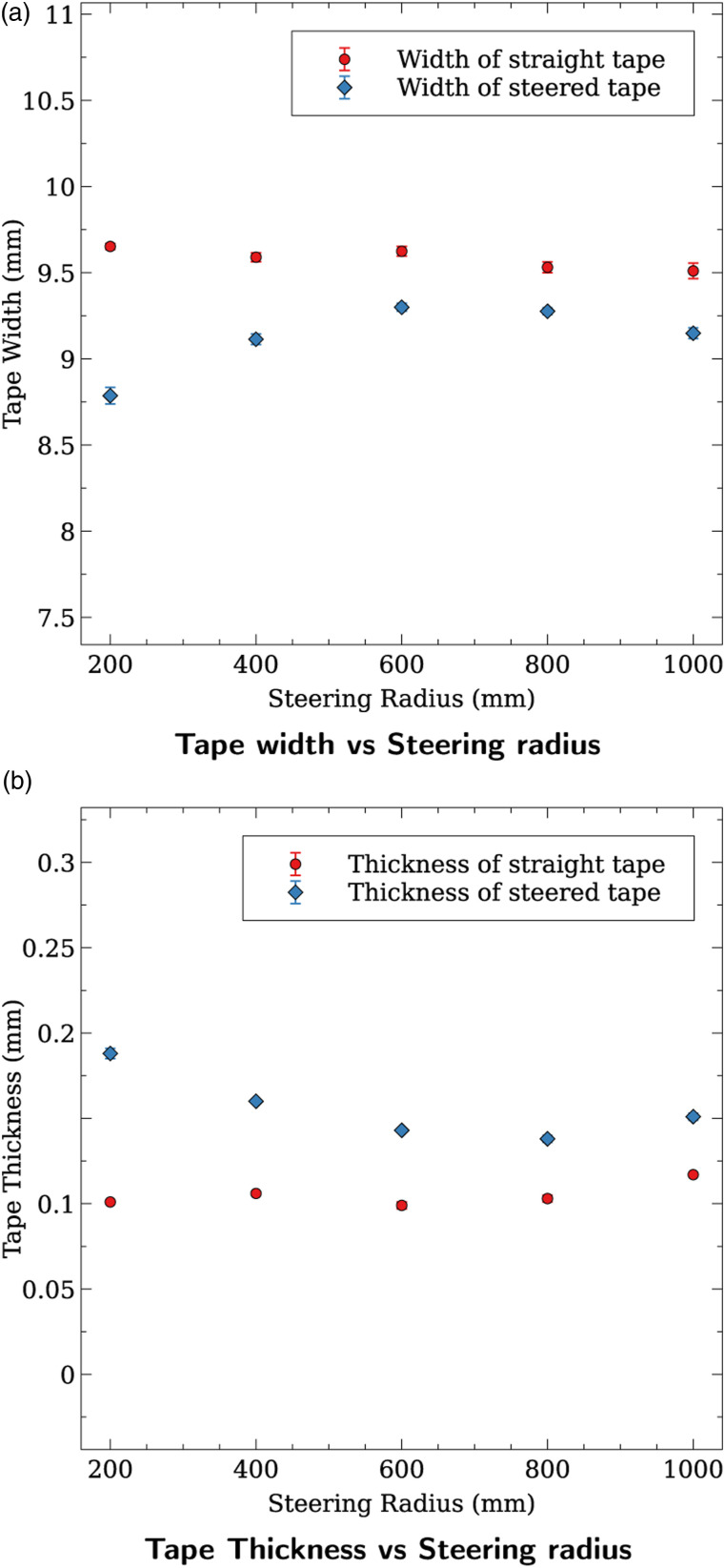
Effect of steering radius on the geometry of the tape.

The summary of the results obtained by measuring the width and thickness of the steered tape in comparison with the straight tape is shown in Table 2. The width and thickness of the straight portion of the tapes remained almost constant. The width of the steered portion was found to be consistently lower than straight portion. A similar observation was made in a previous study by Clancy et al.^15^ This is primarily due to the steering which causes the fibers in the tape to move more closer to and even slip on the top of each other. This causes the width of the tape to be lesser than what it would be when the tape was laid down in a straight line. Figure 13 shows two distinct regions of variation in the width and thickness of the tape. The first region is for samples with steering radii of 1000 *mm*, 800 *mm*, and 600 *mm* (sample number 1 to 9 in Table 1) where the widths and thicknesses of these radii remain very close to each other. A second trend observed in the samples 10 to 15 (Table 1) with sharper steering radii of 400 *mm* and 200 *mm*. In the latter case, the widths of the steered tapes are further reduced (Figure 13(a)) and their thicknesses increase more sharply (Figure 13(b)) because of the tape buckling and tape folding that occurred at these radii. Analysis of the cross-sectional area of tapes (multiplication of width by thickness) shows a general trend on the effect of steering. Comparing the straight tapes cross-sectional areas with those of steered ones under same process conditions shows that steering increases the tape cross-sectional area. The smaller the steering radii, the bigger the increase in the cross-sectional area would be. This trend may be attributed to the defect (void, buckling, and tape folding) formation during steering.

**Table 2.** Change in radii results.

Measurement	Radii (mm)	Straight (mm)	SE(±)	Steered (mm)	SE(±)
Width	1000	9.511	0.045	9.149	0.031
	800	9.532	0.031	9.276	0.020
	600	9.625	0.028	9.300	0.023
	400	9.590	0.026	9.114	0.031
	200	9.653	0.017	8.787	0.048
Thickness	1000	0.117	0.001	0.151	0.002
	800	0.103	0.002	0.138	0.002
	600	0.099	0.002	0.143	0.001
	400	0.106	0.001	0.160	0.002
	200	0.101	0.001	0.188	0.003

SE: Standard Error.

#### Effect of laydown speed

**Figure 14.**
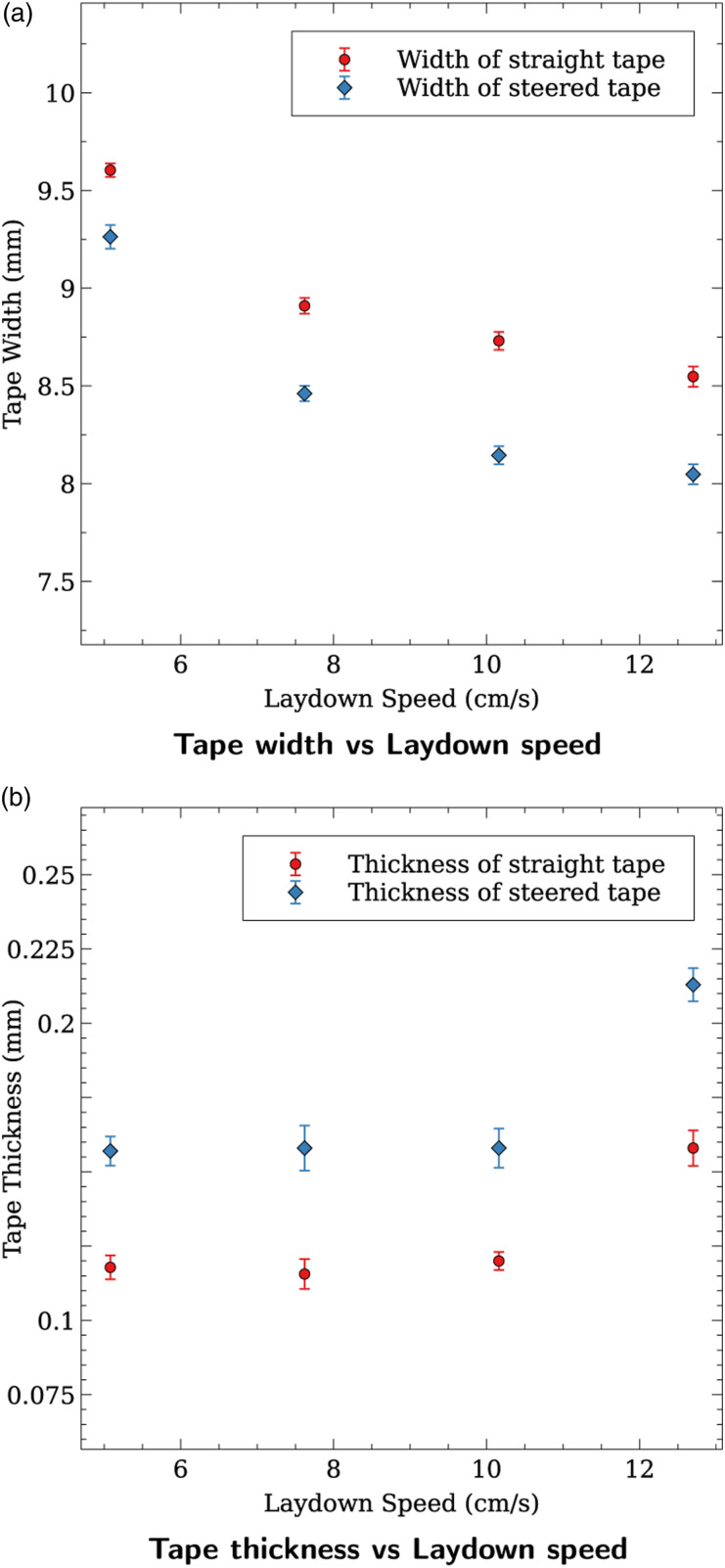
Effect of laydown speed on the geometry of the tape at a steering radius of 400 *mm*.

The summary of the results obtained by measuring the widths and thicknesses of tapes with varying laydown speed is shown in Table 3. The width of the both the straight and the steered tape reduces as the laydown speed of the AFP increases. This is because the resin has less time under the consolidation roller to be squeezed and spread in the width direction. Steering also further decreases the width of the laid down tape. However, in the study by Clancy et al.^15^ on steering of thermoplastic tape with a Laser-assisted ATP, the width of the straight tape remained unchanged up to a laydown speed of 16.67 *cm*/*s*. For the steered portion of the tape, the width of the tape remained almost constant until the laydown speed of 10 *cm*/*s* but had a sharp decrease at the speed of 16.67 *cm*/*s*.^15^ The thickness almost remains constant for the straight region despite the width of the tape reducing with increasing speed, but there is a 35*%* increase at the speed of 12.7 *cm*/*s* (5*in*/*s*). The thickness of the steered layer is higher than the straight portion at every speed. The variation of the thickness along each of the steered samples is very similar to the straight samples with a sharp increase at the laydown speed of 5in/s. This observation for the steered portion is consistent with the study by Clancy et al.^15^ The variation of width and thickness is visualized in the graphs shown in Figure 14.

**Table 3.** Effect of laydown speed on the geometry of the tape.

Measurement	Speed (cm/s)	Straight (mm)	SE(±)	Steered (mm)	SE(±)
Width	12.17	8.548	0.052	8.048	0.051
	10.16	8.731	0.046	8.145	0.046
	7.62	8.910	0.040	8.461	0.039
	5.08	9.604	0.034	9.263	0.061
Thickness	12.17	0.158	0.006	0.213	0.006
	10.16	0.120	0.003	0.158	0.007
	7.62	0.115	0.005	0.158	0.008
	5.08	0.117	0.004	0.157	0.005

SE: Standard Error.

#### Effect of repass

**Figure 15.**
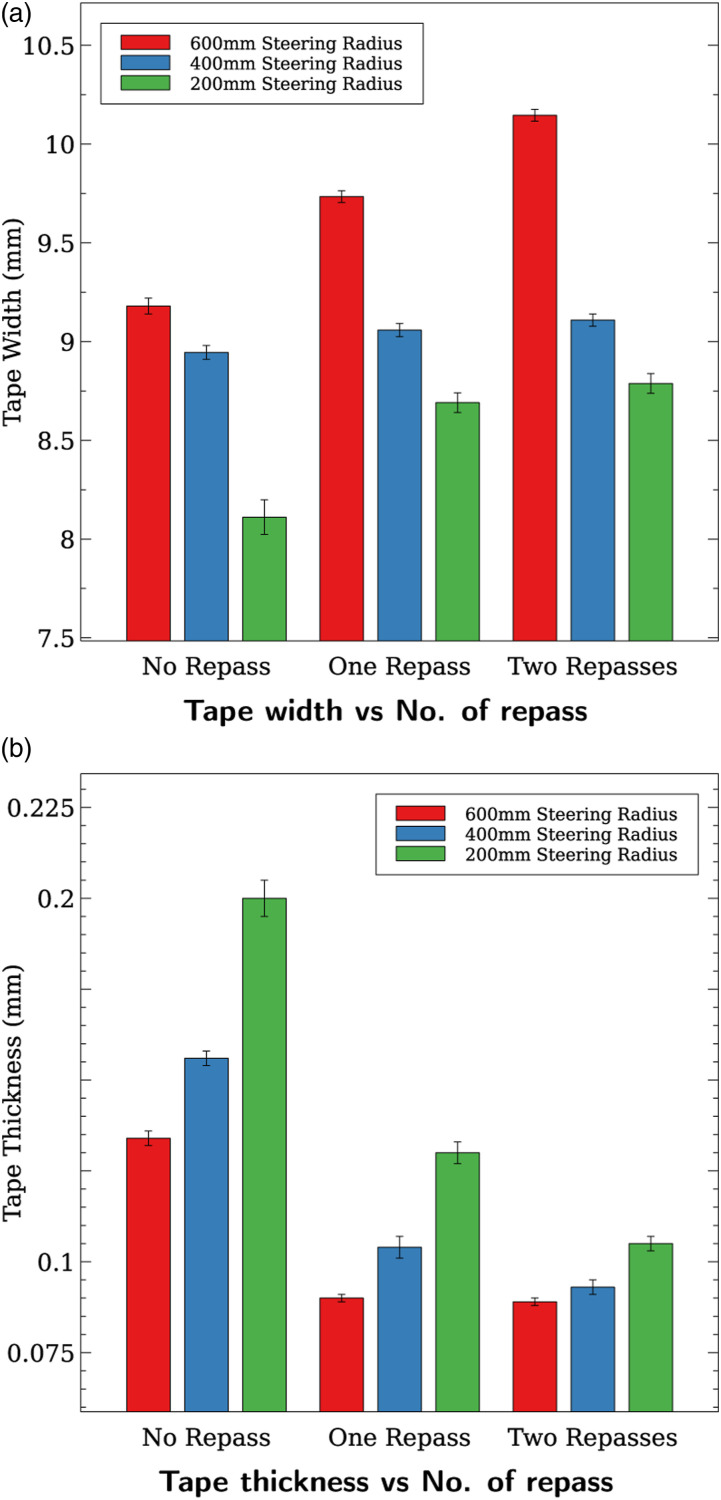
Effect of repass on the geometry of the tape.

During the trials where the laydown speed and steering radius were varied, it was observed that the fiber pull-up due to the buckling of the tape occurred in some of the cases. Repass was applied to the steered tape to see if it could ameliorate these steering-induced defects. The repass was done at same HGT temperature and consolidation force (i.e., 875°*C* and 266.89 *N*). The results of the geometrical analysis done on samples that underwent repass are summarized in Table 4. The first repass increases the width and reduces the thickness. However, the second repass does not lead to a considerable change in the tape’s dimensions in most cases; the change in the width at the steering radius of 600 *mm* is the only exception to this trend. These variations are represented in the plots shown in Figure 15.

**Table 4.** Effect of repass on the geometry of the tape.

Radii (mm)	No. of repass	Width (mm)	SE(±)	Thickness (mm)	SE(±)
600	0	9.180	0.039	0.134	0.002
	1	9.734	0.034	0.090	0.001
	2	10.146	0.033	0.089	0.001
400	0	8.945	0.035	0.156	0.002
	1	9.058	0.033	0.104	0.003
	2	9.109	0.030	0.093	0.002
200	0	8.111	0.088	0.200	0.005
	1	8.691	0.049	0.130	0.003
	2	8.788	0.050	0.105	0.002

SE: Standard Error.

### Microscopic analysis

#### Effect of steering radius

**Figure 16.**
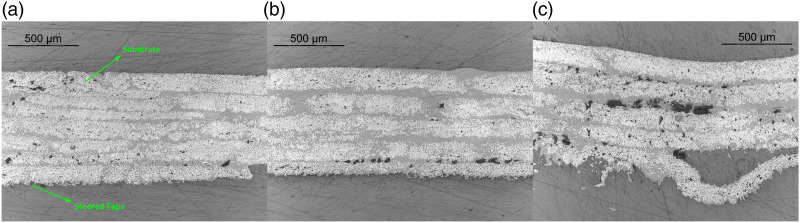
Effect of steering radius. (a) Good bond consolidation at 800 mm radius, (b) voids at interface—400 mm radius, and (c) tape folding at 200 mm radius.

The microscopic analysis was performed on each sample to see how well the bond of the steered layer was consolidated with the substrate and to see how the fibers were distributed. For higher steering radii like 1000 *mm* and 800 *mm*, no buckling or tape folding observed in the visual inspection. This was also reflected in the microscopic analysis showing good consolidation and no voids due to the steering-induced defects as shown in Figure 16(a). However, for smaller steering radii like 400 *mm* and 200 *mm*, there were void formations at the bond line (Figure 16(b)), and in case of the 200 *mm* steering radius, the tape folding can be clearly observed in Figure 16(c).

**Figure 17.**
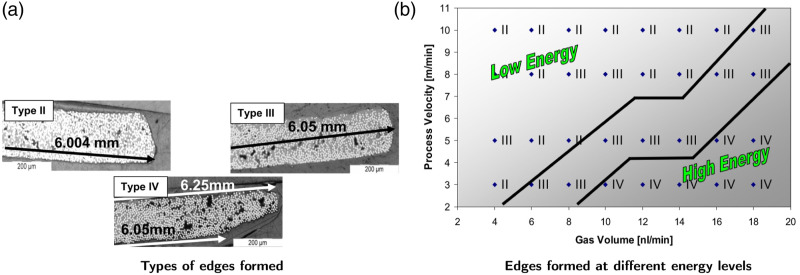
Thermoplastic tape edge formations at various energy levels.^31^

Different types of edge formations were characterized by Lawal et al.^30^ and were also observed by M.A. Khan,^31^ as shown in Figure 17(a). The study by Khan^31^ investigated the edge formations of a thermoplastic tape due to the energy imparted by an HGT. The energy imparted was considered to be a function of the laydown speed and the hot gas flow rate. As seen in the graph shown in Figure 17(b), high speed and low gas flow rates resulted in lower energy being imparted and type II edges were formed. In our study, a similar type II edge was observed at a steering radius of 800 *mm* and speed of 5.08 *cm*/*s* (2*in*/*s*), as shown in Figure 16(a).

#### Effect of laydown speed

**Figure 18.**
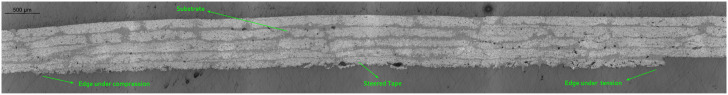
Tape steered at 10.16* cm*/*s* with a radius of 400* mm* (HGT temp 875°*C*; Compaction 266.89 *N*).

During visual inspection is was seen that for the tape steered at 400 *mm* radius at speeds of 10.16 *cm*/*s* (4*in*/*s*) and 12.7 *cm*/*s* (5*in*/*s*), the defects were no longer continuous along the length of the tape, it was intermittent and defects only occupied a small percentage of the length of the tape. When observed under a microscope, the tapes appeared well consolidated and without significant defects at all the speeds tested (Figure 18). This is consistent with the observation made in the Laser-assisted ATP steering study by Clancy *et al.*^15^ Only in the case of 2in/s (Figure 16(b)), some void formation was found on the bond line. Clear conclusions regarding the effect of laydown speed on steered tapes cannot be drawn using microscopic analysis alone, further mechanical testing is required, see the section *Effect of Laydown Speed*.

#### Effect of repass

**Figure 19.**
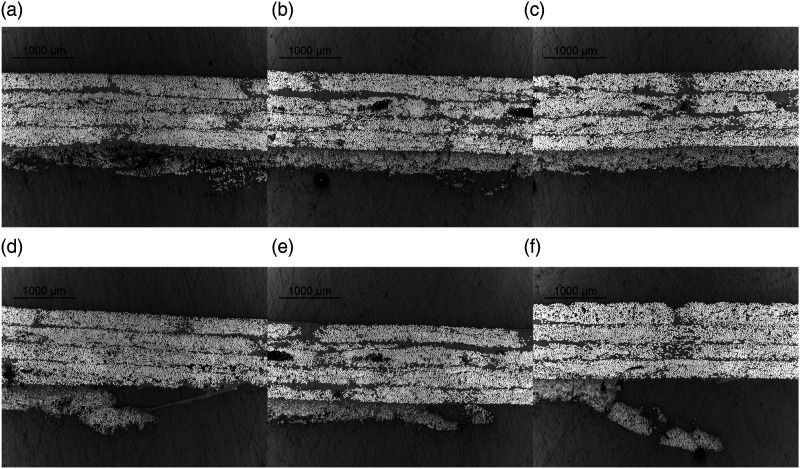
Effect of repass on a steering radius of 400 mm (HGT temp 875°*C*; Compaction 266.89 *N*).

To see if the defects caused by tape steering can be ameliorated, samples of 600 *mm*, 400 *mm*, and 200 *mm* were treated with 1 and 2 repasses. The laydown speed was 5.08 *cm*/*s* (2*in*/*s*) and HGT temperature and compaction force were 875°*C* and 266.89 *N*(60*lbs*), respectively, as per Table 1. Figure 19(a)–(c) shows the effect of repass on a steered tape. As observed during the visual inspection, the buckling defects are smoothened by repass. However, even after two repass treatment, the tape folding is not rectified by repass and may have caused the folded edge to break (Figure 19(f)).

### Mechanical testing

#### Effect of steering radius

**Figure 20.**
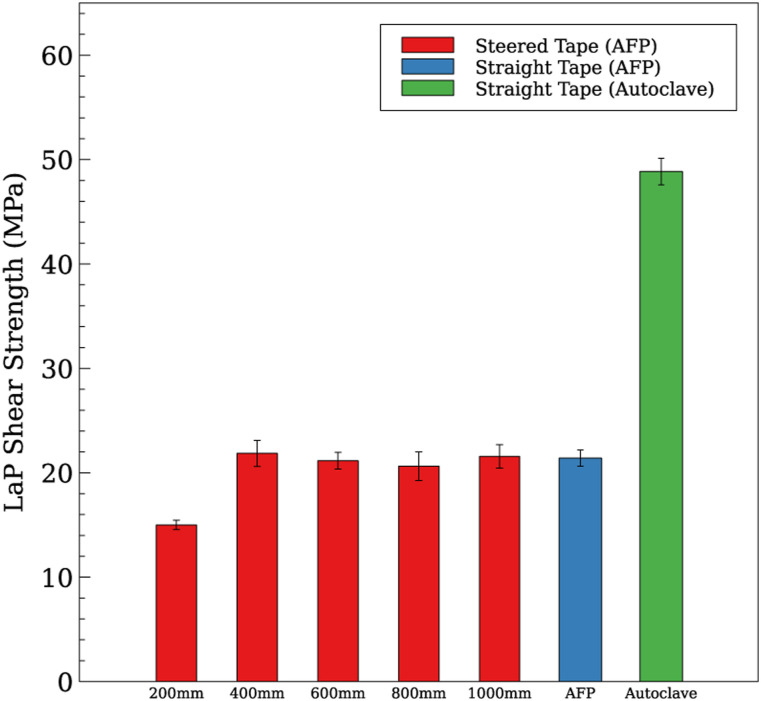
Steering Radius versus Lap Shear Strength (LSS).

Samples of different steering radii ranging from 1000 *mm* to 200 *mm* were manufactured and tested as described in the section *Mechanical Testing*. For each steering radius tested, five samples were manufactured and tested based on the modified lap shear test as explained in the section *Mechanical Testing*. To study the effect of steering radius on the bond strength, all samples were manufactured with a constant laydown speed of 5.08 *cm*/*s* (2*in*/*s*), compaction force of 266.89 *N* (60*lbs*), HGT temperature of 875°*C*, and HGT flow rate of 0.06 *m*^3^/min. To serve as a reference, two sets of straight samples were also manufactured using the AFP, using the same process parameters mentioned above. One set was directly tested using the modified lap shear test (the section *Mechanical Testing*). The other set was post-consolidated using an autoclave as per the procedure described in the section *Reference Sample Manufacturing Using Autoclave* and then tested using the modified lap shear test. The summary of the mean LSS obtained for the samples and their failure modes are shown in Table 5 and represented in graphical form in Figure 20.

**Table 5.** Lap Shear Strength (LSS) results for different steering radii.

Radius (mm)	LSS (MPa)	SE(±) (MPa)	Failure mode
1000	21.57	1.12	LFT
800	20.64	1.37	LFT
600	21.15	0.80	LFT
400	21.86	1.25	LFT
200	15.01	0.45	LFT
Straight tape (in-situ)	21.409	0.77	LFT
Straight tape (autoclave)	48.86	1.27	Cohesive

LFT: Light Fiber Tear; SE: Standard Error.

**Figure 21.**
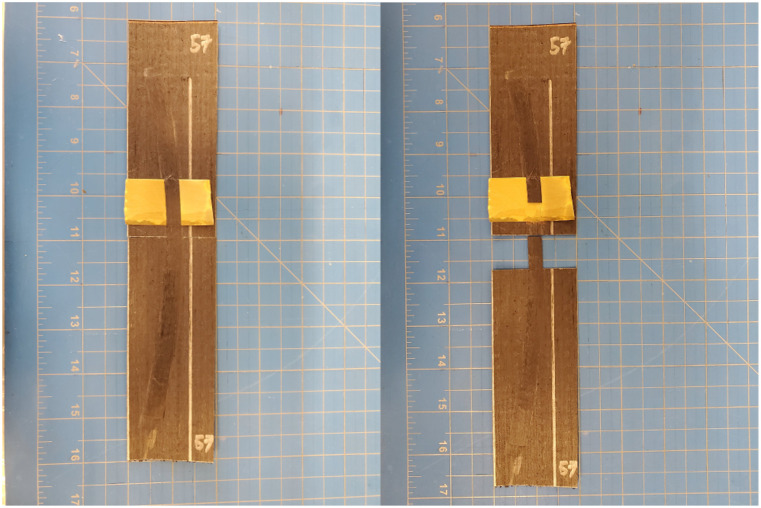
Lap shear test samples at 400 mm steering radius.

The results show that with these process parameters steering at a radii from 1000 *mm* down to 400 *mm* does not affect the bond strength negatively and the LSS of the steered tapes is very comparable to the results obtained for a straight tape (without post-treatment in autoclave). These results are interesting as they indicate that despite some defects observed in 600 *mm* and 400 *mm* tapes, the process parameters used are sufficient to provide a bonding as good as the straight tape made by the AFP alone. However, this is only true until a steering radius of 400 *mm* and there is sharp fall in the bond strength at a steering radius of 200 *mm*. This is mainly due to the prominent tape folding observed at this radius resulting in a poor bond along the outer edge of the tape. Another important observation is that the LSS of samples post-treated in the autoclave (48.86 *MPa*) is more than two times of those made by in-situ consolidation of AFP (21.409 *MPa*). This large difference is due to the fact that during the in-situ consolidation, the molecules of the thermoplastic matrix do not have enough time to diffuse into the substrate and make a perfect bond as is the case for autoclave-treated samples. Figure 21 shows a sample of 400 *mm* steering radius that failed by the light fiber tear failure mode.

#### Effect of laydown speed

**Figure 22.**
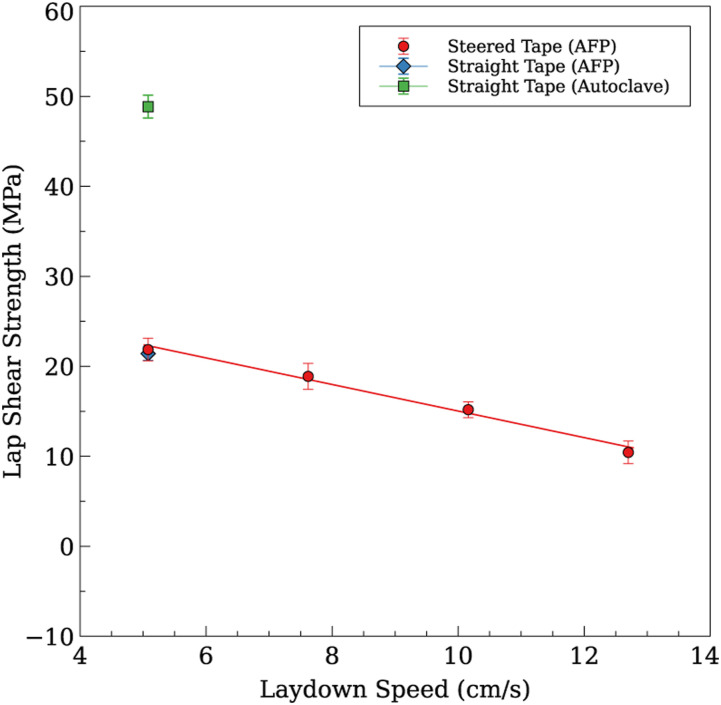
Laydown Speed versus Lap Shear Strength (LSS).

To study the effect of laydown speed on the bond strength, a steering radius of 400 *mm* was chosen and samples were manufactured as described in the section *Mechanical Testing*. A consolidation force of 266.89 *N*(60*lbs*), HGT temperature of 875°*C*, and HGT flow rate of 0.06 *m*^3^/min were kept constant for all the samples manufactured at different laydown speeds. Five samples for each laydown speed were manufactured,^27^ and straight tapes laid down at a speed of 5.08 *cm*/*s*(2*in*/*s*) and then post-consolidated in an autoclave were used as a reference. The summary of the results obtained by testing all the samples of different laydown speeds is shown in Table 6 and represented in graphical form in Figure 22. The test results indicate that the bond strength has an inversely linear relationship with the laydown speed when all the other process parameters are kept constant. This relationship can be observed clearly in Figure 22. This is very similar to what is observed in the case of unidirectional tapes.^32^ This behavior of decreasing bond strength with increasing laydown speed was because the incoming thermoplastic tape did not have enough time under the HGT and compaction roller to melt and consolidate with the substrate. This insufficient consolidation resulted in a poor bonding between the incoming tape and the substrate.

**Table 6.** Lap Shear Strength (LSS) results for different laydown speeds.

Laydown speed (cm/s)	LSS (MPa)	SE(±) (MPa)	Failure mode
5.08	21.86	1.25	LFT
7.62	18.88	1.44	LFT
10.16	15.18	0.88	LFT
12.7	10.43	1.26	LFT
Straight tape (in-situ)	21.409	0.77	LFT
Straight tape (autoclave)	48.86	1.27	Cohesive

LFT: Light Fiber Tear; SE: Standard Error.

#### Effect of substrate angle

To investigate the effect of the substrate angle on the bond strength, substrates were made such that the top layer forms the angles of 0°, 30°, 60°, and 90° with the gauge area of the tape. To test whether steering affects the bond adversely in such cases, samples were made for both straight and steered tapes. Samples were manufactured at a laydown speed of 5.08 *cm*/*s*(2*in*/*s*), under 266.89 *N*(60*lbs*)of consolidation pressure, 875°*C* of HGT temperature, and an HGT flow rate of 0.06 *m*^3^/min. For the steered samples, a radius of 400 mm was chosen as in the section *Effect of Laydown Speed*.

**Figure 23.**
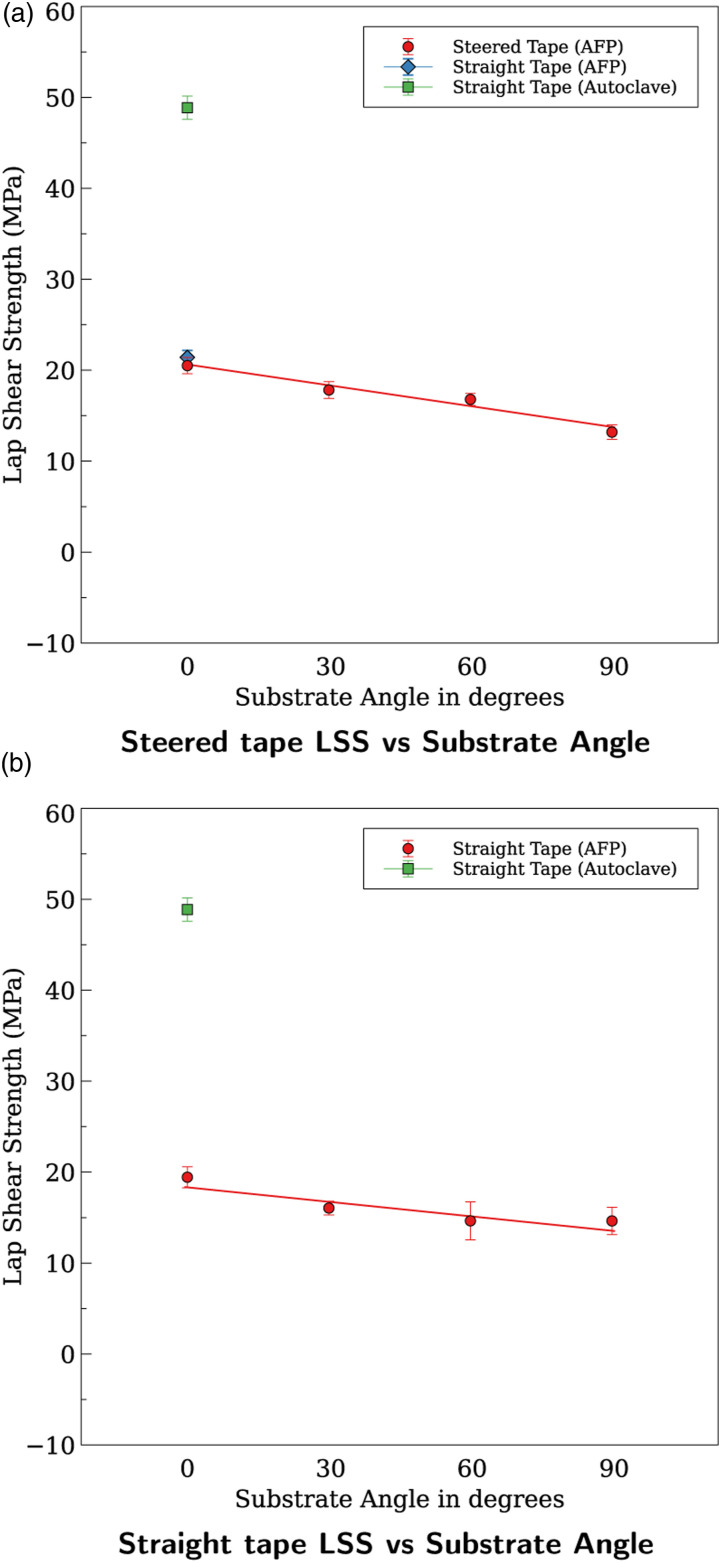
Lap Shear Strength versus Substrate Angle.

The test results obtained for steered and straight tapes with different substrate angles have been summarized in Table 7 and represented in graphical form in Figure 23. The results show a clear inversely linear relationship between the Lap Shear Strength and substrate angle for both the steered and straight tapes. These results agree with another study by Grefe et al.^33^ that investigated the effect of fiber orientation on lap shear strength and found that the highest LSS was at 0° and descended to the lowest at 90°. As it was shown in the section *Effect of Steering Radius*, at the radius of 400 *mm* and above no appreciable difference in the LSS between the straight and the steered tapes was observed. This implies that that one can perform fiber steering on any substrate angle without compromising on the bond strength. However, it should be noted that for radii below 400 *mm*, the effect of the radius will be more dominant due to the tape folding defect causing the bond strength to suffer.

**Table 7.** Lap Shear Strength (LSS) results for different substrate angles.

Type	Substrate angle	Mean (MPa)	SE(±) (MPa)	Failure mode
Steered	0°	20.501 5	0.900	LFT
	30°	17.823 1	0.920	LFT
	60°	16.786 6	0.657	LFT
	90°	13.190 1	0.789	LFT
Straight	0°	19.434 7	1.157	LFT
	30°	16.045 0	0.760	LFT
	60°	14.637 0	2.085	LFT
	90°	14.628 8	1.509	LFT
Straight tape (autoclave)	0°	48.86	1.27	Cohesive

LFT: Light Fiber Tear SE: Standard Error.

## Conclusion

In this study, the ability of HGT-assisted AFP to steer 6.35 *mm* (0.25*in*) wide CF/PEEK tapes was investigated. The effect of different parameters including steering radius, laydown speed, number of repasses, and substrate angle on the geometry and lap shear strength was investigated.

The tapes were steered at different radii and no considerable steering-induced defects were observed until the radius of 400 *mm*. At a radius of 400 *mm*, some tape buckling and some hints of tape folding were observed. The smallest radii of 200 *mm* suffered the most defects due to the steering as both tape buckling and tape folding were dominant. It was found that steering-induced defects are continuous along the tape when laid down at a speed of 5.08 *cm*/*s* (2*in*/*s*) and 7.62 *cm*/*s* (3*in*/*s*). At higher speeds of 10.16 *cm*/*s* (4*in*/*s*) and 12.7 *cm*/*s* (5*in*/*s*), the defects were intermittent. Optical microscopy showed that the outer edges of tapes that were steered at 400 *mm* and 200 *mm* radii were poorly bonded due to the tape folding. These tapes also experienced some fiber pull up due to the tape buckling. Repasses were attempted on these tapes to resolve these defects, and it was found that a single repass was sufficient to smoothen most of the fiber pull up. However, the tape folding was not fixed even after two repasses and resulted in the breaking of the folded edge of the tape.

A specially devised lap shear test was used to test the bond strength of the steered CF/PEEK tapes with a CF/PEEK substrate that was also manufactured by the HGT-assisted AFP. Some of the AFP-made samples were post-consolidated in the autoclave to provide a reference value for lap shear strength. Testing the bond strength of different steering radii and comparing it with straight tapes laid down with identical process parameters yielded interesting results. The test results indicate that bond strength of the steered tapes up to a radius of 400 *mm* was as good as the straight tapes. However, at the radius of 200 *mm*, there was a sharp fall in the bond strength. The severe tape folding prevalent at this radius is believed to be the primary cause of this reduction in the bond strength. Another important observation was that the bond strength of AFP in-situ consolidated samples was roughly less than half those post-consolidated in the autoclave.

It was also found that the lap shear strength decreases linearly with increasing laydown speed by testing four different laydown speeds at a steering radius of 400 *mm*. The tapes steered at speeds of 10.16 *cm*/*s* (4*in*/*s*) and 12.7 *cm*/*s* (5*in*/*s*) showed poor bond strength despite having much lower steering-induced defects. This highlighted the importance of choosing proper process parameters to allow sufficient heat transfer to take place to melt the thermoplastic resin completely and bond well with the substrate. The bond strength at higher speeds may be improved by using a higher gas flow rate or higher hot gas temperature. The effect of substrate angle was also investigated and the results showed that the bonding was strongest at the substrate angle of 0° and weakest at 90°.

This study gives us a clear understanding of the ability of the HGT AFP process to perform fiber steering. However, a more detailed study is required to fully understand the role of substrate fiber angle on a steered tape. Future work in this field would be to tackle other issues in the manufacturing VAT laminates such as gaps and overlaps.
